# The Impact of Circulating Tumor Cells on Venous Thromboembolism and Cardiovascular Events in Bladder Cancer Patients Treated with Radical Cystectomy

**DOI:** 10.3390/jcm9113478

**Published:** 2020-10-28

**Authors:** Michael Rink, Sabine Riethdorf, Hang Yu, Mara Kölker, Malte W. Vetterlein, Roland Dahlem, Margit Fisch, Klaus Pantel, Armin Soave

**Affiliations:** 1 Department of Urology, University Medical Center Hamburg-Eppendorf, D-20246 Hamburg, Germany; yuhang.seu@outlook.com (H.Y.); m.koelker@uke.de (M.K.); m.vetterlein@uke.de (M.W.V.); r.dahlem@uke.de (R.D.); m.fisch@uke.de (M.F.); a.soave@uke.de (A.S.); 2 Institute of Tumor Biology, University Medical Center Hamburg-Eppendorf, D-20246 Hamburg, Germany; s.riethdorf@uke.de (S.R.); pantel@uke.de (K.P.)

**Keywords:** bladder cancer, urothelial carcinoma, circulating tumor cell, venous thromboembolism, cardiovascular event, radical cystectomy

## Abstract

Background: Cancer is a relevant risk factor for venous thromboembolism (VTE). Circulating tumor cells (CTC) are associated with an increased risk of VTE in breast cancer. In addition, circulating cell-free nucleic acids have been associated with cardiovascular events (CVE). Objective: To investigate the association of CTC status and the risk of VTE as well as CVE in urothelial carcinoma of the bladder (UCB) patients treated with radical cystectomy (RC). Methods: We collected data of 189 UCB patients treated with RC at our institution. Blood samples were acquired preoperatively and analyzed for CTC using the CellSearch^®^ system. Thirty-day postoperative complications were extracted from digital charts and graded according to the Clavien–Dindo classification (CDC). Moreover, each patient’s individual Comprehensive Complication Index^®^ (CCI^®^) was calculated. Results: CTC were present in 43 patients (22.8%). Overall, six patients experienced VTE (3.2%) and eight patients (4.2%) experienced CVE. There was no association of VTE or CVE according to CTC status. In total, 168 patients (89%) experienced a total of 801 complications, of which the majority was classified as “minor” (CDC grade ≤ IIIa; 79%). There was no association between CTC status and any grade of a complication or CCI^®^. Presence of CTC was associated with more aggressive clinicopathological UCB features. Conclusions: The overall rate of VTE and CVE was low in our study. Presence of CTC was neither associated with an increased risk of VTE nor CVE in UCB patients treated with RC. According to this study, CTC are not a qualified biomarker for individualized thromboprophylaxis management in these patients.

## 1. Introduction

Radical cystectomy (RC) with or without perioperative chemotherapy is the gold standard treatment for muscle-invasive and recurrent refractory non-muscle invasive urothelial carcinoma of the bladder (UCB) [1]. Despite significant improvements in operative and perioperative management, RC denotes major pelvic surgery associated with high rates of intra- and postoperative complications [2]. Venous thromboembolism (VTE) and cardiovascular events (CVE) are relevant, potentially fatal complications frequently following major pelvic surgeries, including RC [3]. Indeed, apart from the surgical procedure, several cancers have been identified as an independent risk factor for thromboembolic events [4]. Tumor cells can activate hemostasis or fibrinolytic systems by variable mechanisms, including the production of procoagulant or pro-aggregating factors such as tissue factor cancer procoagulant and/or plasminogen activator inhibitors as well as the release of pro-inflammatory and proangiogenic cytokines [5,6]. Hence, peri- and postoperative pharmacological thromboprophylaxis is strongly recommended by international guidelines after RC [7].

Circulating tumor cells (CTC) are malignant epithelial cells, which represent an occult cancer burden in the peripheral blood stream. The presence of CTC is associated with unfavorable clinical outcomes in UCB patients treated with RC [8,9]. In metastatic breast cancer patients, the presence of CTC was found to be associated with an increased risk of VTE [10]. In addition, circulating cell-free nucleic acids (cf-NAs) in human plasma have been associated with cardiovascular disease [11]. Indeed, the introduction of a biomarker that identifies surgically treated patients with an additional cancer-related increased VTE or CVE risk would be of great clinical interest, as the clinical management and duration of perioperative thromboprophylaxis could be individually tailored. At present, the association of CTC and VTE or CVE has not been investigated in UCB. We hypothesized that UCB patients treated with RC and the presence of CTC harbor an increased risk for both endpoints.

## 2. Materials and Methods

### 2.1. Study Population

We prospectively collected the data of 219 UCB patients treated with RC and bilateral pelvic lymph node dissection at our institution between January 2007 and April 2014 and some other data were published before [8,12,13]. We previously described indication for RC, preoperative patient workup, surgical intervention, and pathological workup [13]. A detailed description is provided as Supplementary Material. To investigate the unbiased morbidity outcomes from purely surgically treated patients, patients undergoing neoadjuvant chemotherapy were not eligible. The study was approved by the local ethics committee (Nb. PV3779).

### 2.2. Blood Sample Analyses and CTC Investigations

In general, one to seven days prior to RC, all the patients received blood tests of various laboratory parameters. Blood coagulation tests included thrombocyte count (Mrd/L), INR (Quick), activated partial thromboplastin time (aPTT; seconds), thrombin time (TT; seconds), and fibrinogen measurement (g/L). The C-reactive protein (CRP) is a systemic inflammatory response marker that is linked to tumorbiological features and outcome in UCB [14] and is associated with an increased risk of cancer-associated VTE [4]. The CRP assay was not standardized over time, but when available, high-sensitivity CRP was measured according to a method previously described [15]. CTC were detected using the CellSearch^®^ system as previously described [8] (see also: Supplemental Material).

### 2.3. Extraction and Grading of Complications

Postoperative complications of 189 patients were extracted from digital charts. Any complication within the first 30 days after RC was recorded for each patient according to the predefined complication catalogue described by Vetterlein et al. [2]. Each complication was graded according to the validated and adapted Clavien–Dindo classification (CDC) [16,17]. In addition, we calculated the 30-day Comprehensive Complication Index^®^ (CCI^®^) [18] for each patient using the online calculator provided on http://cci.assessurgery.com. Based on the CDC, the CCI^®^ represents the perioperative course in a scale ranging from 0 (uneventful course) to 100 (death). Cause of death was determined by the treating physician, by chart review corroborated by death certificates, or by death certificates alone [19].

### 2.4. Statistical Analysis

The primary study endpoint was VTE defined as clinically apparent or diagnostic-confirmed deep venous thrombosis and/or pulmonary embolism. The coprimary endpoint was CVE defined as any myocardial infarction, new onset angina pectoris, cerebrovascular events, transient ischemic attack, or peripheral arterial ischemia.

The patient cohort was stratified according to CTC status (CTC presence vs. no presence). Clinical, pathological, and laboratory parameters were compared according to the CTC status as well as the coprimary endpoints. Due to the limited number of overall events, no further analyses according to the CTC number were employed. The clinical and laboratory parameters were also compared between patients experiencing one of the primary endpoints and those who did not. In addition, the overall preoperative morbidity burden as reflected by the CDC and CCI^®^ was analyzed and compared between both CTC groups.

All blood coagulation laboratory values were analyzed as continuous variables. CRP was analyzed as a continuous variable and dichotomized in low or high CRP-values. The cut-off for high CRP was 5 mg/L [15].

Continuous variables were reported as means and standard deviations or medians and interquartile ranges (IQRs). The Kolmogorov–Smirnov test was used to assess the normal distribution of variables. Categorical variables were presented as frequencies and proportions. For comparisons between the groups, a Chi-square test, Fisher’s exact test, Student’s t-test, and Mann–Whitney U test were used as appropriate. All reported *p*-values are two-sided, and statistical significance was set at *p* < 0.05. All statistical tests were performed with IBM SPSS Statistics 25 (IBM Corp., Armonk, NY, USA).

## 3. Results

### 3.1. Descriptive Characteristics of the Study Cohort

Clinicopathologic characteristics of the entire study cohort and stratified according to CTC status are presented in Table 1. The median age was 68 years and 137 (73%) patients were male. Overall, 43 (23%) patients had the presence of CTC with an average of 10 ± 33 cells/7.5 mL blood (median: 1; range: 1–163). Presence of CTC was associated with a higher rate of positive soft tissue surgical margin (STSM), presence of lymphovascular and microvascular invasion, and administration of adjuvant chemotherapy (all *p*-values ≤ 0.047).

### 3.2. Primary Endpoint Analyses

In total, six (3.2%) VTE occurred in all patients, among which four patients (2.1%) had deep venous thrombosis, one patient (0.5%) had pulmonary embolism, and one patient (0.5%) had both deep venous thrombosis and pulmonary embolism, respectively. In total, eight patients (4.2%) experienced CVE (Table 2).

Three of 43 (7.0%) patients with presence of CTC experienced a VTE and 1/43 (2.3%) patients experienced a CVE, respectively; both were not significantly different from patients without CTC presence (Table 2). In patients with CTC presence, the aPTT was longer (30.1 vs. 28.9 s, *p* = 0.016) and the fibrinogen level was higher (4.72 vs. 4.12 g/L, *p* = 0.007) compared to patients without CTC presence. Table 3 summarizes the results of clinical and laboratory index parameters for the subgroups of patients with VTE and CVE, compared to patients without any of these events. The patient cohort was homogenously distributed without statistically significant differences in age, gender, duration of RC (OR-time), American Society of Anesthesiologists (ASA), Eastern Cooperative Oncology Group (ECOG), or Age-adjusted Charlson comorbidity index (ACCI) scores between patients with or without any VTE or CVE, respectively. There was no statistically significant difference in any laboratory value for all analyzed comparisons.

### 3.3. Evaluation of All 30-Day Postoperative Complications

In total, 801 complications were captured in 168 patients (89%), which translated into a median of 4 (IQR 2–6) complications per patient. The most common complications types were infectious (18.9%), gastrointestinal (18.6%), and genitourinary (18.1%) complications, respectively (Supplementary Table S1). Table 4 presents a summary of the 30-day postoperative complications graded by CDC for the entire study cohort and stratified by CTC status. In total, seventeen patients (8.9%) experienced the most severe complications (CDC grade ≥ IIIb) requiring another intervention under general anesthesia and an additional two patients (1.1%) had fatal complications and died during the early postoperative course (CDC grade V). The majority of complications, however, were graded “minor” (CDC grade I: 14.8%; CDC grade II: 60.8%; CDC grade IIIa: 3.2%). There was no difference among any grade of complications between CTC positive and negative patients.

The median CCI^®^ considering all complications for the entire cohort was 27.2 (IQR: 18.4;34.3). The median CCI^®^ for patients with presence of CTC was lower compared to patients without CTC presence, which did not reach statistical significance (25.7 vs. 27.2; *p* = 0.54). In the subgroup of patients experiencing a VTE, the median CCI^®^ was higher compared to patients without a VTE (35.6 (IQR: 32.2;60.2) vs. 25.7 (17.3;33.2); *p* = 0.002). In the subgroup of patients experiencing a CVE, the median CCI^®^ also exceeded the CCI^®^ for patients without a CVE (44.6 (IQR: 34.0;70.8) vs. 25.7 (17.3;33.2); *p* = 0.03).

## 4. Discussion

We found that presence of CTC was not associated with an increased risk of VTE in our cohort. Thus, we have to reject our null hypothesis. In general, several malignancies including UCB are highly associated with a tumor-induced increased risk for VTE [4]. The pathogenesis of cancer-associated VTE is multifactorial and likely involves multiple overlapping pathways including Virchow’s triad, surgery- or chemotherapy-induced endothelial damage, cancer-related immobility, and cancer-induced hypercoagulability [4]. Malignant tumor cells can activate a direct coagulation pathway, inhibit fibrinolytic activity, and induce inflammatory responses in various complex ways, respectively, of which each single or a combination may trigger VTE. Thus, it intuitively seemed reasonable to investigate the association between CTC and VTE, as today, in the absence of reliable evidence, all UCB patients receive the same recommendation regarding medical antithrombotic prophylaxis [7]. A biomarker, therefore, would be helpful for a personalized risk assessment and individually tailored decision-making regarding risk of VTE and duration of prophylaxis. The VTE rate in our cohort (3.2%) is in congruence with the incidence after RC depending on preexisting risk factors in the literature (2.6–11.6%) [20]. Our findings are in contrast to a study in metastatic breast cancer patients, in which CTC detected by the CellSearch^®^ system were significantly associated with an increased risk of VTE [10]. Differences between the study by Mego et al. and ours include the underlying tumor entity, status of disease, and type of treatment among others, which may explain differential findings. Especially in early UCB stages, CTC occur in very low concentrations in the peripheral blood [21]. Next to the cancer itself, in UCB, the highest risk of VTE is reported in patients undergoing RC, due to numerous surgical factors. We, therefore, investigated potential confounding clinical and surgical factors including age, preexisting morbidity, and operation time and found no difference between patients experiencing a VTE and those who did not in all parameters. Indeed, the limited numbers of patients with CTC presence and primary endpoint events may have masked significant associations in our study.

Despite cancer-induced VTE being substantially more pervasive, cardiovascular disease due to the presence of malignancy, chemotherapeutic drugs, and cancer-related comorbidities represents a relevant clinical issue. In fact, according to a recent population-based study among various cancer entities, UCB patients have the highest risk of dying from a cardiovascular disease [22]. Circulating cf-NAs have been reported to offer promising new insights in the regulation of normal physiological functions and in the development of pathological alterations. Thus, they may allow diagnosis and monitoring for various cardiovascular diseases [11]. In UCB, next to cell-free tumor DNA and RNA, CTC are another encouraging real-time blood-based biomarker that reflects potentially occult metastatic disease [9] and better predicted overall survival compared to other circulating biomarkers including cf-DNA [23]. We, therefore, investigated the associations of CTC and CVE in our study. We found that CTC presence was not associated with CVE in our cohort. Although the CVE rate in our study is in line with previous reports [2,24], the absolute event rate is limited, which may have concealed significant associations and also interfered further detailed analyses for this heterogeneous group of events. In fact, CTC and cf-NAs do not indiscriminately reflect the same source of origin and therefore, may predict variable endpoints. CTC usually represent epithelial cells that can be distinguished from other blood cells by various technical mechanisms and therefore, identify malignant tumor burden originating from the primary tumor or its metastasis. In contrast, cf-NAs in cancer patients do not only originate only from tumor cells, but also from cells of the tumor microenvironment and non-cancer cells from various parts of the body [25]. In patients with cardiovascular disease, an increased rate of cf-NAs may be released into circulation from dying cells during normal cellular turnover or active cell death, especially in the case of acute ischemic vascular events. In addition, the surgical procedure itself also may increase the cf-NA rate after RC. A detailed analysis of the origin of cf-NA would be warranted in UCB patients to discriminate between cancer-derived cf-NAs and other origins. Unfortunately, we were unable to include cf-NA analyses in this study to investigate differential findings compared to CTC.

We analyzed various laboratory parameters that are commonly used in clinical routine to investigate their association with CTC status and both study endpoints, respectively. We found a higher median fibrinogen level in patients with presence of CTC, without any association to VTE or CVE. Various cancers including UCB may activate the coagulation/fibrinolysis system, with fibrinogen being an acute phase protein performing a relevant step in the coagulation process. It has been reported that increased preoperative plasma fibrinogen levels are associated with adverse tumorbiological features [26] and a risk factor for 1-year mortality after RC in UCB patients [27]. In congruence, the increased fibrinogen level in our study may reflect the more aggressive tumorbiological features in CTC-positive patients, including higher rates of pT4 and pN+ disease, positive surgical margins as well as the presence of LVI and microvessel invasion, although not all of these parameters were statistically significantly different from CTC-negative patients. Moreover, we found an elevated median of aPTT in patients with CTC presence. The aPTT is a coagulation test measuring the general speed at which blood clots, by means of the intrinsic and common pathways of coagulation, among others, monitor the treatment effects of heparin. Shortened aPTT times may indicate consumption and have been reported to be associated with an increased risk of thrombosis [28]. The oncological meaning of aPTT remains ambiguous, especially in urological malignancies [29]. Since aPTT was not associated with VTE, CVE, or any other clinicopathologic variable, we rate our finding as rather unspecific to avoid overestimation. The independent oncological prognostic relevance of both laboratory parameters in UCB patients treated with RC might be interesting and therefore, the object of future studies in larger cohorts, but this was not the subject of this study. Unfortunately, we were unable to include other popular biomarkers in our study such as D-Dimer, a potentially predictive marker for VTE risk [4].

Our data confirm the overall tremendous risk of morbidity after RC. Almost 90% of patients had a median of four complications within 30-days after surgery. This rate is substantially higher compared to previous reports [24], which may be due to our meticulous assessment of 30-day complications after RC according to the revised Martin criteria of the updated European Association of Urology (EAU) guidelines using a digital patient chart. We used a refined analysis of complications compared to previous studies, as we analyzed complications using the CDC and CCI^®^ score, respectively. Next to the CDC grading of only the highest complication during a specified time frame, we also mirrored a patient’s cumulative morbidity burden using the CCI^®^ to reduce underreporting [2]. In congruence with the existing literature, the broad majority of complications were classified as “minor” according to CDC and only 9% had a complication CDC grade ≥ IIIb. As the quality and quantity of a complication are paramount, the CCI^®^ provides a more intuitive reflection of the perceived overall morbidity. Indeed, it is debatable whether every single undesirable event should be considered a complication. Still, we found that with patients experiencing a VTE or CVE, both had significantly higher CCI^®^ scores compared to patients without these complications. While this primarily indicates a larger amount and/or more severe complications in these patients, it also underscores the utmost importance of preoperative screening for cardiovascular diseases in UCB patients [22] as well as various perioperative approaches to minimizing the VTE risk [3]. In contrast, we found no difference in CDC or CCI^®^ between patients with or without CTC presence. Although this intuitively is not surprising, it is an important piece of information considering the fact that the presence of CTC is a powerful predictor for inferior outcomes [8] and thus, CTC-positive patients may be candidates for adjuvant chemotherapy [13].

Although to our knowledge, this is the first study investigating the association of CTC with VTE and CVE in UCB patients treated with RC, this study is not devoid of limitations. First and foremost are limitations inherent to the overall sample size and event rates. Some clinical and laboratory factors (e.g., family history of thrombosis or lupus anticoagulant) were not routinely assessed in all patients, which may have influenced study endpoints. Obviously, complications do also occur >30 days after RC. We chose 30 days to be able to extract the most possible granular data from our digitalized charts and to mitigate information loss during retrospective data extraction due to recall bias and documentation quality [2]. In addition, VTE and CVE usually occur early during the postoperative course and the majority of these events are captured in the first 3 weeks post-surgery [30,31]. Nevertheless, a reevaluation of the association of CTC with both endpoints in the intermediate- and long-term post-RC interval may be of great interest. In addition, perioperative administration of low-weight molecular heparin on a regular basis may have masked significant associations between CTC and outcomes. All patients in our study were treated by open RC, which may have influenced results, as differences in the risk of VTE and CVE between open and laparoscopic or robotic-assisted RC have been reported [7]. If CTC dissemination is also influenced by the type of RC currently remains unclear. In addition, our study did not include patients treated with neoadjuvant chemotherapy. Indeed, complication rates as well as CTC measures may vary in patients undergoing neoadjuvant chemotherapy. To improve the generalizability of our findings, larger studies including patients with neoadjuvant chemotherapy and different types of surgery are desirable. Finally, our results rely on investigations using the CellSearch^®^ system, which still remains the only validated, FDA-approved technology for the detection of CTC. Nevertheless, there are different commercially available platforms with variable CTC detection rates [9]. While malignant CTC in the peripheral blood stream may be missed by the CellSearch^®^ platform, it also still needs to be proven that every epithelial cell detected with this system truly is a malignant CTC.

## 5. Conclusions

According to our study, CTC are not associated with an increased risk of VTE or CVE in UCB patients treated with RC. As a consequence, CTC do not seem to represent a biomarker for individually tailored medical prophylaxis to minimize the risk of cancer-associated VTE or CVE in patients after RC. However, the limited number of patients with presence of CTC as well as VTE and CVE events in our study warrant reevaluation in a larger patient cohort.

## Figures and Tables

**Table 1:** Descriptive clinicopathological characteristics of 189 UCB patients who underwent radical cystectomy and bilateral lymphadenectomy.

	All *N* = 189	CTC Negative *N* = 146	CTC Positive *N* = 43	*p*-Value
Age (years; median (IQR))	68 (58;75)	68 (59;75)	70 (57;75)	0.810
Gender (*n*;%)				0.333
male	137 (72.5)	103 (70.5)	34 (79.1)	
female	52 (27.5)	43 (29.5)	9 (20.9)	
ASA (*n*;%)				0.613
1	2 (1.0)	2 (1.4)	0(0)	
2	95 (50.3)	76 (52.1)	19 (44.2)	
3	89 (47.1)	66 (45.2)	23 (53.5)	
4	3 (1.6)	2 (1.4)	1 (2.3)	
ECOG-PS (*n*; %)				0.169
0	169 (89.4)	133 (91.1)	36 (83.7)	
≥1	20 (10.6)	13 (8.9)	7 (16.3)	
ACCI * (*n*; %)				0.904
0	5 (2.6)	4 (2.7)	1 (2.3)	
1	18 (9.5)	13 (8.9)	5 (11.6)	
2	25 (13.2)	20 (13.7)	5 (11.6)	
≥3	85 (45.0)	68 (46.6)	17 (39.5)	
Intraoperative blood loss (mL; median (IQR))	500 (200;800)	500 (200;800)	550 (300;750)	0.865
Pathological Tumor Stage (*n*; %)				0.090
pT0	19 (10.1)	18 (12.3)	1 (2.3)	
pTa	11 (5.8)	8 (5.5)	3 (7.0)	
pTis	10 (5.3)	9 (6.2)	1 (2.3)	
pT1	18 (9.5)	16 (11.0)	2 (4.7)	
pT2	45 (23.8)	32 (21.9)	13 (30.2)	
pT3	61 (32.3)	48 (32.9)	13 (30.2)	
pT4	25 (13.2)	15 (10.3)	10 (23.3)	
Combined disease stage (*n*; %)				0.296
Localized (≤pT2)	103 (54.5)	83 (56.8)	20 (46.5)	
Advanced (≥pT3)	86 (45.5)	63 (43.2)	23 (53.5)	
Pathological Tumor Grade (*n*; %)				0.283
No grading (pT0)	16 (8.5)	15 (10.3)	1 (2.3)	
G2	17 (9.0)	13 (8.9)	4 (9.3)	
G3	156 (82.5)	118 (80.8)	38 (88.4)	
Concomitant carcinoma in situ (*n*)				0.585
Absent	123 (65.1)	97 (66.4)	26 (60.5)	
Present	66 (34.9)	49 (33.6)	17 (39.5)	
Lymph node status (*n*; %)				0.135
pN0	132 (69.8)	106 (72.6)	26 (60.5)	
pN+	57 (30.2)	40 (27.4)	17 (39.5)	
Number of lymph nodes removed (median (IQR))	15 (10;22)	15 (10;22)	14 (10;22)	0.427
Margin status (*n*; %)				**0.014**
R0	163 (86.2)	131 (89.7)	32 (74.4)	
R+	26 (13.8)	15 (10.3)	11 (25.6)	
Lymphovascular invasion (*n*; %)				**0.041**
Absent	130 (68.8)	106 (72.6)	24 (55.8)	
Present	59 (31.2)	40 (27.4)	19 (44.2)	
Microvascular invasion (*n*; %)				**0.047**
Absent	163 (86.2)	130 (89.0)	33 (76.7)	
Present	26 (13.8)	16 (11.0)	10 (23.3)	
Adjuvant Chemotherapy (*n*; %)				**0.007**
No	137 (72.5)	113 (77.4)	24 (55.8)	
Yes	52 (27.5)	33 (22.6)	19 (44.2)	

Abbreviations: UCB—urothelial carcinoma of the bladder; CTC—circulating tumor cells; IQR—interquartile range; ASA—American Society of Anesthesiologists; ECOG-PS—Eastern Cooperative Oncology Group Performance Status; ACCI—Age-adjusted Charlson comorbidity index. * ACCI was not available for 56 (29.6%) patients. Bold characters indicate statistical significance.

**Table 2:** Comparative analysis of venous thromboembolism, cardiovascular events, and laboratory index parameters of 189 UCB patients treated with radical cystectomy and bilateral lymphadenectomy stratified by CTC status.

	All *N* = 189	CTC Negative *N* = 146	CTC Positive *N* = 43	*p*-Value
Venous Thromboembolism (*n*; %)				0.132
No	183 (96.8)	143 (97.9)	40 (93.0)	
Yes	6 (3.2)	3 (2.1)	3 (7.0)	
Deep vein thrombosis	4 (2.1)	2 (1.4)	2 (4.7)	
Pulmonary embolism	1 (0.5)	1 (0.7)	0(0)	
Both	1 (0.5)	0 (0)	1 (2.3)	
Cardiovascular Events (*n*; %)				0.685
No	181 (95.8)	139 (95.2)	42 (97.7)	
Yes	8 (4.2)	7 (4.8)	1 (2.3)	
Myocardial Infarction	1 (0.5)	1 (0.7)	0 (0)	
Angina pectoris	2 (1.1)	2 (1.4)	0 (0)	
CVA/TIA	3 (1.6)	2 (1.4)	1 (2.3)	
Angina pectoris and Myocardial Infarction	1 (0.5)	1 (0.7)	0 (0)	
Angina pectoris and CVA/TIA	1 (0.5)	1 (0.7)	0 (0)	
C-reactive protein (mg/L; median (IQR))	1 (1;11.5)	1 (1;11)	7 (1;14)	0.137
Low (*n*; %)	114 (60.3)	93 (63.7)	21 (48.8)	0.08
High (*n*; %)	75 (39.7)	53 (36.3)	22 (51.2)	
Thrombocyte (Mrd/L; median)	282	277	298	0.213
Quick (INR; median)	0.97	0.97	0.98	0.442
APTT (s; median)	29.13	28.86	30.07	**0.016**
TT (s; median)	17.60	17.68	17.31	0.145
Fibrinogen (g/L; median)	4.25	4.12	4.72	**0.007**

Abbreviations: UCB—urothelial carcinoma of the bladder; CTC—circulating tumor cells; CVA—cerebrovascular accident; TIA—transient ischemic attack; IQR—interquartile range; INR—international normalized ratio; APTT—activated partial thromboplastin time; TT—thrombin time. Bold characters indicate statistical significance.

**Table 3:** Analyses of clinical and laboratory index parameter in patients with venous thromboembolic or cardiovascular events after radical cystectomy for UCB treatment.

Parameter	All *N* = 189	Subgroup	All Others	*p*
		**VTE (6 pts)**	**No VTE (183 pts)**	
Age (year; median (IQR))	68 (58;75)	73 (53;76)	68 (60;75)	0.796
Male gender (*n*; %)	137 (72.5)	5 (3.6)	132 (96.4)	0.889
ASA (*n*; %)				0.981
1	2 (1.0)	0 (0)	2 (1.1)	
2	95 (50.3)	3 (50.0)	92 (50.3)	
3	89 (47.1)	3 (50.0)	86 (47.0)	
4	3 (1.6)	0 (0)	3 (1.6)	
ECOG ≥ 1 (*n*; %)	20 (10.6)	0 (0)	20 (100)	0.506
ACCI * (*n*; %)				0.355
0	5 (2.6)	0 (0)	5 (2.7)	
1	18 (9.5)	2 (33.3)	16 (8.7)	
2	25 (13.2)	0 (0)	25 (13.7)	
≥3	85 (45)	4 (66.7)	81 (44.3)	
OR-Time (median; IQR)	273 (225;335)	244 (189;316)	275 (225;335)	0.292
C-reactive protein (mg/L; median (IQR))	1 (1;12)	6 (1;35)	1 (1;11)	0.518
Low (*n*; %)	114 (60.3)	3 (50.0)	111 (60.7)	0.920
High (*n*; %)	75 (39.7)	3 (50.0)	72 (39.3)	
Thrombocyte (Mrd/L; median)	282	323	281	0.295
Quick (INR; median)	0.97	1.07	0.97	0.344
APTT (s; median)	29.10	29.70	29.10	0.654
TT (s; median)	17.60	17.60	17.60	0.996
Fibrinogen (g/L; median)	4.25	4.47	4.25	0.665
		**CVE (8 pts)**	**No CVE (181 pts)**	
Age (year; median (IQR))	68 (58;75)	75(55;82)	68(60;75)	0.333
Male gender (*n*; %)	137 (72.5)	5(62.5)	132(72.9)	0.809
ASA (*n*; %)				0.889
1	2 (1.0)	0(0)	2(1.1)	
2	95 (50.3)	5(62.5)	90(49.7)	
3	89 (47.1)	3(37.5)	86(47.5)	
4	3 (1.6)	0(0)	3(1.7)	
ECOG ≥ 1 (*n*; %)	20 (10.6)	2(10)	18(90)	0.443
ACCI * (*n*; %)				0.397
0	5 (2.6)	0(0)	5(2.8)	
1	18 (9.5)	2(25)	16(8.8)	
2	25 (13.2)	0(0)	25(13.8)	
≥3	85 (45)	5(62.5)	80(44.2)	
OR-Time (median; IQR)	273 (225;335)	283(259;354)	275(225;335)	0.592
C-reactive protein (mg/L; median (IQR))	1 (1;12)	12(3;20)	1(1;11)	0.068
Low (*n*; %)	114 (60.3)	2(25)	112(61.9)	0.086
High (*n*; %)	75 (39.7)	6(75)	69(38.1)	
Thrombocyte (Mrd/L; median)	282	299	281	0.606
Quick (INR; median)	0.97	0.97	0.97	0.991
APTT (s; median)	29.10	28.70	29.10	0.668
TT (s; median)	17.60	17.40	17.60	0.654
Fibrinogen (g/L; median)	4.25	4.95	4.22	0.106

Abbreviations: UCB—urothelial carcinoma of the bladder; VTE—venous thromboembolism; IQR—interquartile range; ASA—American Society of Anesthesiologists; ECOG-PS—Eastern Cooperative Oncology Group Performance Status; ACCI—Age-adjusted Charlson comorbidity index; INR—international normalized ratio; APTT—activated partial thromboplastin time; TT—thrombin time; CVE—cardiovascular Events. * ACCI was not available for 56 (29.6%) patients.

**Table 4:** CDC grading and CCI^®^ score of 30-day postoperative complications in 189 UCB patients who underwent radical cystectomy between 2007 and 2013.

	All *N* = 189	CTC Negative *N* = 146	CTC Positive *N* = 43	*p*-Value
CDC grading				0.996
0	21 (11.1)	17 (11.6)	4 (9.3)	
Grade I	28 (14.8)	22 (15.1)	6 (14.0)	
Grade II	115 (60.8)	87 (59.6)	28 (65.1)	
Grade IIIa	6 (3.2)	5 (3.4)	1 (2.3)	
Grade IIIb	12 (6.3)	9 (6.2)	3 (7.0)	
Grade IVa	5 (2.6)	4 (2.7)	1 (2.3)	
Grade IVb	0 (0)	0 (0)	0 (0)	
Grade V	2 (1.1)	2 (1.4)	0 (0)	
CCI (median (IQR))	27.2 (18.4;34.3)	27.2 (17.3;34.3)	25.7 (20.9;30.8)	0.537

Abbreviations: CDC—Clavien–Dindo classification; CCI—Comprehensive Complication Index; UCB—urothelial carcinoma of the bladder; CTC—circulating tumor cells.

## Supplementary Materials

Supplementary Materials can be found at https://www.mdpi.com/2077-0383/9/11/3478/s1. Supplementary Material and Methods; Supplemental Table S1. Frequencies, proportions, therapeutic management, and grading of perioperative 30-day complications in 189 patients who underwent radical cystectomy between January 2007 and December 2013.
