# Supplementary material for: The Impact of Circulating Tumor Cells on Venous Thromboembolism and Cardiovascular Events in Bladder Cancer Patients Treated with Radical Cystectomy
Source: J Clin Med. 2020 Oct 28;9(11):3478. doi: 10.3390/jcm9113478 (PMC7692134; doi:10.3390/jcm9113478)
Supplement: Supplementary file 1 [file jcm-09-03478-s001.zip › jcm-975073-supplemental table-track changes.pdf]

Journal of Clinical Medicine

MDPI

Supplemental Table 1. Frequencies, proportions, therapeutic management, and grading of perioperative 30-day complications in 189 patients who underwent radical cystectomy between January 2007 and December 2013.

	CDC grading	Management	Number of complications	Proportion (*N*=189)
**Gastrointestinal: 149 complications (18.6%)* in 107 patients**				
Ileus (paralytic)	I	Conservative; cessation of oral intake and i.v. fluid support	10	5.3%
	II	Medical management beyond laxatives, NG tube placement, or intravenous nutrition management indicated	9	4.8%
	IIIa	Replacement of nasogastric tube	2	1.1%
Small bowel obstruction (mechanical)	IIIb	Laparotomy	0	--
Constipation	I	Conservative; laxatives, i.v. fluid support	30	15.9%
Clostridium difficile colitis	II	Antibiotic treatment	2	1.1%
Gastrointestinal bleeding	I	Conservative; clinical observation or diagnostic evaluation only	0	--
	II	Blood transfusion	0	--
	IIIb	Esophagogastroduodenoscopy	0	--
Emesis	I	Conservative; antiemetics and i.v. fluid support	48	25.4%
Anastomotic bowel leak	IIIb	Laparotomy	2	1.1%
Diarrhea (≠ Clostridium difficile-associated)	I	Conservative; antidiarrhoeals, i.v. fluid support, electrolytes	46	24.3%
**Infectious: 151 complications (18.9%)* in 113 patients**				
**Fever of unknown origin**	**II**	**Conservative; antipyretics, antibiotic treatment**	**6**	3.2%
Bacteriuria (> 10^5 cfu/ml; asymptomatic)	I	Conservative; no antibiotic treatment	39	20.6%
Urinary tract infection (> 10^5 cfu/ml; symptomatic)	II	Antibiotic treatment	101	53.4%
Abscess	II	Antibiotic treatment	1	
	IIIa	Incision and drainage	0	--
	IIIb	Incision and drainage + general anesthesia	0	--

Sepsis (SIRS in response to infectious process)	II	Antibiotic treatment, supportive care	2	1.1%
	IVa	Septic single organ dysfunction, ICU	0	--
	IVb	Septic multi-organ dysfunction, ICU	0	--
	V	Septic multi-organ dysfunction leading to death	0	--
Pyelonephritis	II	Antibiotic treatment	0	--
Diverticulitis	II	Antibiotic treatment	1	0.5%
Gastroenteritis	II	Conservative; antiemetics, antibiotic treatment	1	0.5%
Cholecystitis	IIIb	Cholecystectomy	0	--

**Wound: 35 complications (4.4%)* in 30 patients**				
**Wound seroma**	**I**	**Conservative; clinical observation or diagnostic evaluation only**	**0**	--
Wound infection (SSI)	II	Antibiotic treatment	7	3.7%
Wound dehiscence (fascia intact)	I	Conservative; clinical observation or diagnostic evaluation only, reinforced adhesive skin closure	18	9.5%
	IIIb	Secondary surgical closure	8	4.2%
Fascial dehiscence/evisceration	IIIb	Secondary surgical closure	2	1.1%

Table 3 (Continued)

	**CDC grading**	**Management**	Number of complications	Proportion (*N*=189)
**Genitourinary**: **145 complications (18.1%)* in 104 patients**				
Acute kidney injury	I	Conservative; i.v. fluid support, diuretics	5	2.6%
	II	Medical treatment and nutrition support	1	0.5%
	IVa	Dialysis	0	--
Hydronephrosis/ureteral obstruction (new onset)	I	Conservative; clinical observation or diagnostic evaluation only	45	23.8%
Urinary leak/urinoma	I	Conservative; clinical observation or diagnostic evaluation only, deferred Foley catheter extraction	3	1.6%
	IIIa	Drainage	0	--
	IIIb	Ureter re-implantation/retrograde ureteral stenting	0	--

Urinary retention	I	Conservative; clinical observation or diagnostic evaluation only, re-placement of Foley catheter (neobladder/pouch)	2	1.1%
Parastomal hernia	I	Conservative; clinical observation or diagnostic evaluation only	0	--
	IIIb	Laparotomy and surgical revision	0	--
Urostomy ischemia	I	Conservative; clinical observation or diagnostic evaluation only	0	--
	IIIb	Laparotomy and surgical revision	0	--
Hematuria	I	Conservative; clinical observation or diagnostic evaluation only	89	47.1%

**Cardiac: 42 complications (5.2%)* in 33 patients**				
**Arrhythmia**	**I**	**Conservative; clinical observation**	**4**	2.1%
	II	Medical cardioversion	4	2.1%
	IIIb	Pacemaker, cardioversion	4	2.1%
Myocardial infarction	IVa	Coronary angiography and stent implantation, ICU	2	1.1%
Hypertension (new onset)	II	Antihypertensives	13	6.9%
(Acute) congestive heart failure	IVa	Coronary angiography, ICU	4	2.1%
Angina (pectoris)	I	Conservative; clinical observation or diagnostic evaluation only	4	2.1%
Hypotension	II	Medical treatment	7	3.7%

**Pulmonary: 16 complications (2.0%)* in 12 patients**				
**Atelectasis**	**II**	**Continuous positive airway pressure, physiotherapy**	**0**	--
Pneumonia	II	Antibiotic therapy	8	4.2%
Respiratory distress/dyspnea	I	Oxygen, physiotherapy	3	1.6%
Pneumothorax	I	Conservative; clinical observation or diagnostic evaluation only	1	0.5%
	IIIa	Chest tube	0	--
Pleural effusion	I	Conservative; clinical observation or diagnostic evaluation only	4	2.1%
	IIIa	Chest tube	0	--

Table 3 (Continued)

	CDC grading	Management	Number of complications	Proportion (*N*=189)
**Bleeding: 54 complications (6.7%)* in 48 patients**				
Anemia requiring transfusion	II	Blood transfusion	38	20.1%
Postoperative bleeding other than gastrointestinal	I	Conservative; clinical observation or diagnostic evaluation only	2	1.1%
	II	Blood transfusion/fibrinogen	0	--
	IIIa	Suture ligation	0	--
	IIIb	Laparotomy and surgical revision	1	0.5%
Wound hematoma	I	Conservative; clinical observation or diagnostic evaluation only	13	6.9%
**Thromboembolic: 6 complications (0.7%)* in 5 patients**				
**Deep vein thrombosis**	**II**	**Anticoagulation**	**4**	2.1%
Pulmonary embolism	I	Conservative; clinical observation or diagnostic evaluation only	2	1.1%
	II	Anticoagulation	0	--
	IVa	ICU, anticoagulation	0	--
Superficial phlebitis	II	Conservative; heparin ointment	0	--
**Neurological: 47 complications (5.9%)* in 45 patients**				
**Peripheral neuropathy**	**I**	**Conservative; clinical observation or diagnostic evaluation only**	**33**	17.5%
CVA/TIA	II	Antiplatelets, anticoagulation	4	2.1%
Delirium/Agitation	II	Antipsychotics	10	5.3%
Vertigo	II	Medical therapy	0	--
Loss of consciousness/Syncope	I	Conservative; clinical observation or diagnostic evaluation only	0	--
Seizure	II	Medical therapy	0	--
**Intraoperative:** 1 complication (0.1%)* in 1 patient**				
**Vascular injury**	**--**	**--**	**0**	--
Bowel injury	--	Revision surgery	1	0.5%
Retained foreign body	--	--	0	--
**Miscellaneous: 155 complications (19.4%)* in 98 patients**				
**Psychological illness**	**I**	**Conservative; psycho-oncological support**	**5**	2.6%
Dermatitis	I	Ointment	6	3.2%

Acidosis	I	Conservative; medical therapy, electrolytes	2	1.1%
Decubitus ulcer	I	Conservative	14	7.4%
Lymphocele	I	Conservative; clinical observation or diagnostic evaluation only	11	5.8%
	IIIa	Drainage	1	0.5%
Dehydration	I	Conservative; i.v. fluid support	0	--
Edema	I	Conservative; medical therapy	60	31.7%
Hypokalemia	I	Conservative; medical therapy	7	3.7%
Other rare complications	I	Conservative	49	25.9%

* The percentage refers to the proportion of all 801 complications.

** The CDC does not apply to intraoperative complications. Thus, no grading system was used.

CDC=Clavien-Dindo classification; cfu=colony-forming units; CVA=cerebrovascular accident; ICU, intensive care unit; i.v. = intravenous; SIRS=systemic inflammatory response syndrome; SSI, surgical site infection; TIA, transient ischemic attack.

Percentages may not add up to 100%, as they are rounded.

© 2020 by the authors. Submitted for possible open access publication under the terms and conditions of the Creative Commons Attribution (CC BY) license (http://creativecommons.org/licenses/by/4.0/).
