# Supplementary material for: The Impact of Circulating Tumor Cells on Venous Thromboembolism and Cardiovascular Events in Bladder Cancer Patients Treated with Radical Cystectomy
Source: J Clin Med. 2020 Oct 28;9(11):3478. doi: 10.3390/jcm9113478 (PMC7692134; doi:10.3390/jcm9113478)
Supplement: Supplementary file 1 [file jcm-09-03478-s001.zip › jcm-975073-supplementary material and methods-track changes.pdf]

Journal of Clinical Medicine

Supplementary Material and Methods

Study Population

RC indications were muscle invasive UCB or recurrent, refractory Ta, T1 or carcinoma in situ (CIS). Preoperative staging for distant metastasis included cross-sectional imaging of the abdomen/pelvis and thorax. We excluded patients with metastatic UCB at preoperative staging not undergoing RC (n=6), patients with a history of any other malignancy (n=4), male patients with known or suspected coincidental prostate cancer (n=11) and patients with missing data or follow-up (n=6), respectively. In addition, we excluded patients with previous VTE and/or ongoing treatment with low weight molecular heparin for any reason (n=3). None of the patients had a known thrombophilic disease.

CTC investigations

CTC were detected using the CellSearch® system as previously described[1]. In brief, preoperative blood samples (7.5 mL) were obtained on the day before RC in CellSave tubes (Janssen) and analyzed within 96h after collection (median 24 h; interquartile range [IQR]: 24–48 h). Antibodies directed against CK 8, 18 and 19 were used to detect epithelial cells among the cells captured by EpCAM antibodies. For negative selection of leukocytes, a cluster of differentiation (CD)- 45 antibody was used. Nuclei were counterstained with 4,6-diamidino-2-phenylindole. After enrichment and immunochemical staining, immunomagnetically labelled cells were kept in a strong magnetic field and scanned using the CellSpotter™ analyzer (Janssen). Image galleries were assessed manually for CTC according to previously reported criteria[2].

Surgical intervention

All patients underwent open RC using a Bookwalter® table-fixed surgical retractor system. RC included the bladder, distal ureters, prostate and seminal vesicles in men. In women, large anterior pelvectomy was performed including hysterectomy, bilateral adnexectomy, removal of the anterior vagina and the urethra. In general, during pelvic lymph node dissection all fatty and lymphatic tissue was removed from the bifurcation of the common iliac vessels to include all of the distal common iliac, external iliac, obturator and hypogastric nodes on either side. The choice of urinary diversion technique was individually based on patient preference and tumor stage. Generally, we considered continent diversions (i.e., orthotopic neobladder or cutaneous Mainz Pouch I) only for patients with localized disease. Patients with locally advanced tumors or those with significant comorbidity typically underwent ileal conduit or cutaneous ureterostomy. Perioperative management has been reported in detail previously[3]. All patients received low-weight molecular heparin prophylaxis (Enoxaparin 40mg/0.4ml) starting the evening prior surgery and continuing for four weeks after discharge.

Pathological Evaluation

As previously described[1, 4], tumor stage and nodal status were evaluated according to the Tumor-Node-Metastasis (TNM) system. Tumour grade was assessed according to the 1998 World Health Organization (WHO) grading system. Concomitant CIS was defined as the presence of CIS in conjunction with another tumour other than CIS alone. Lymphovascular invasion (LVI) was defined as the unequivocal presence of tumour cells within an endothelium lined space without an underlying muscular wall. A positive soft tissue surgical margin (STSM) was defined as the presence of tumor at inked areas of soft tissue on the RC specimen.

References:

[1] Rink M, Chun FK, Dahlem R, Soave A, Minner S, Hansen J, et al. Prognostic Role and HER2 Expression of Circulating Tumor Cells in Peripheral Blood of Patients Prior to Radical Cystectomy: A Prospective Study. Eur Urol. 2012;61:810-7.

[2] Riethdorf S, Fritsche H, Muller V, Rau T, Schindibeck C, Rack B, et al. Detection of circulating tumor cells in peripheral blood of patients with metastatic breast cancer: A validation study of the CellSearch system. Clin Cancer Res. 2007;13:920-8.

[3] Vetterlein MW, Klemm J, Gild P, Bradtke M, Soave A, Dahlem R, et al. Improving Estimates of Perioperative Morbidity After Radical Cystectomy Using the European Association of Urology Quality Criteria for Standardized Reporting and Introducing the Comprehensive Complication Index. Eur Urol. 2019.

[4] Rink M, Chun FKH, Minner S, Friedrich M, Mauermann O, Heinzer H, et al. Detection of circulating tumour cells in peripheral blood of patients with advanced non-metastatic bladder cancer. BJU International. 2011;107:1668-75.

© 2020 by the authors. Submitted for possible open access publication under the terms and conditions of the Creative Commons Attribution (CC BY) license (http://creativecommons.org/licenses/by/4.0/).
